# State-of-the-Art and Next Generation Intra-Articular Implantable Biosensors for Osteoarthritis: From Analytical Limits to Operational Stability

**DOI:** 10.3390/bios16050283

**Published:** 2026-05-14

**Authors:** Abdullateef Gbolahan Olayiwola, Albina Abdossova, Daniele Tosi, Gorka Orive, Zhe Liu, Cevat Erisken

**Affiliations:** 1 Department of Chemical and Materials Engineering, School of Engineering and Digital Sciences, Nazarbayev University, 010000 Astana, Kazakhstan; abdullateef.olayiwola@nu.edu.kz (A.G.O.); albina.abdossova@nu.edu.kz (A.A.); 2 Department of Electrical and Computer Engineering, School of Engineering and Digital Sciences, Nazarbayev University, 010000 Astana, Kazakhstan; daniele.tosi@nu.edu.kz; 3 Basque Sustainable Pharmacy and Biotherapy Research Group, School of Pharmacy, University of the Basque Country (UPV/EHU), 01006 Vitoria-Gasteiz, Spain; gorka.orive@ehu.eus; 4 Bioaraba Health Research Institute, 01009 Vitoria-Gasteiz, Spain; 5 University Institute for Regenerative Medicine and Oral Implantology—UIRMI (UPV/EHU-Fundación Eduardo Anitua), 01007 Vitoria-Gasteiz, Spain; 6 Academy of Medical Engineering and Translational Medicine, Tianjin University, Tianjin 300072, China; zheliu@tju.edu.cn

**Keywords:** osteoarthritis, osteochondral degeneration, biosensors, osteochondral biomarkers, implantable biosensors, electrochemical biosensors, optical biosensors, synovial fluid

## Abstract

Osteoarthritis (OA) and osteochondral degeneration present a significant clinical burden characterized by the complex interplay of extracellular matrix degradation and chronic inflammation. While biochemical profiling has matured, a critical translational gap remains in transitioning from benchtop assays to systems capable of continuous, intra-articular monitoring. This review provides a comprehensive synthesis of experimentally validated biosensing technologies, including optical, electrochemical, and piezoelectric Quartz Crystal Microbalance (QCM) platforms, evaluated through the lens of sensing architecture, biomarker specificity, and matrix compatibility. Our analysis reveals that while optical sensors offer superior sensitivity, electrochemical platforms show the greatest promise for miniaturized, implantable integration. However, a pivot in the field is identified: the primary bottleneck has shifted from analytical detection limits to operational stability within the hostile synovial environment. Current research is largely restricted to single-analyte detection in simplified media, failing to address the multifactorial nature of OA. We propose that the next generation of osteochondral diagnostics must prioritize multiplexed arrays, mechanically compliant architectures, and machine-learning-assisted signal processing. By bridging these engineering frontiers, biosensors will evolve from passive diagnostic tools into intelligent, personalized platforms for real-time disease management.

## 1. Introduction

The osteochondral (OC) unit of the knee comprises articular cartilage and subchondral bone, connected through a specialized, gradient interface that exhibits distinct physicochemical and biological transitions [1,2]. This functional interface enables biomechanical coupling and biochemical signaling necessary for load distribution and low-friction joint movement [3,4]. Disruption of this system is a major driver of osteoarthritis (OA), a progressive disease affecting more than 500 million people worldwide. Beyond its clinical impact, OA also represents a substantial socioeconomic burden, emphasizing the need for improved strategies for early detection and intervention [5,6,7,8,9].

OA pathogenesis involves complex dysregulation of cartilage and subchondral bone characterized by extracellular matrix (ECM) degradation, abnormal subchondral remodeling, and persistent low-grade inflammation [7,8]. Proteolytic enzymes, particularly matrix metalloproteinases (MMPs) and aggrecanases, contribute to degradation of the collagen–proteoglycan framework, while pro-inflammatory cytokines such as interleukin-1β (IL-1β) and tumor necrosis factor-α (TNF-α) further amplify tissue degeneration [9,10]. Despite this well-established molecular complexity, current diagnostic approaches remain largely reactive rather than preventive. Conventional imaging modalities, including radiography and Magnetic Resonance Imaging (MRI), primarily detect late-stage structural alterations, whereas traditional biochemical assays provide only static measurements of a highly dynamic joint environment [11]. This limitation restricts early detection and longitudinal monitoring of disease progression. Although emerging biomarkers such as cartilage oligomeric matrix protein (COMP), C-terminal telopeptide of type II collagen (CTX-II), and matrix metalloproteinase-3 (MMP-3) demonstrate strong diagnostic potential, their low concentrations and temporal variability within the joint continue to limit clinical utility. These challenges highlight the need for integrated sensing platforms capable of real-time and sensitive biomarker detection.

Biosensors have emerged as promising platforms for the sensitive, selective, and real-time monitoring of osteochondral biomarkers. By integrating biorecognition elements with electrochemical, optical, and flexible electronic transducers, these systems offer opportunities for miniaturized diagnostics capable of operating within the chemically complex joint environment. However, despite substantial advances in analytical performance, most biosensing platforms remain at the proof-of-concept stage with limited validation under physiologically relevant conditions.

This review provides a critical synthesis of osteochondral biosensing technologies by integrating biomarker biology, sampling constraints, and sensor architecture. Unlike previous reviews that address these aspects independently, this work emphasizes their interdependence in determining clinical applicability. Particular focus is placed on implantability, intra-articular performance, operational stability, and translational readiness, with the aim of identifying current limitations and outlining future design priorities for next-generation osteochondral biosensing systems.

## 2. Literature Search Strategy

This review presents a narrative synthesis of the current state of biosensing technologies for osteochondral monitoring. Relevant literature was identified through major scientific databases, including PubMed, Scopus, and Web of Science, using keywords such as “osteoarthritis biomarkers,” “biosensors,” “intra-articular sensing,” “implantable biosensors,” and “synovial fluid.” Priority was given to recent studies and experimentally validated platforms reporting analytical performance, sensing mechanisms, biomarker relevance, and translational potential. Studies focusing exclusively on unrelated diagnostic applications or lacking sufficient methodological and analytical detail were excluded. Additional references were selected based on relevance to biomarker biology, sampling environments, sensor engineering, and clinical applicability. Although this review does not follow a formal systematic protocol, efforts were made to minimize selection bias through the inclusion of representative studies spanning electrochemical, optical, piezoelectric, and emerging biosensing platforms from multiple peer-reviewed sources.

## 3. Soluble and EV Biomarkers

Soluble biomarkers provide a molecular snapshot of the dynamic processes underlying osteochondral degeneration and osteoarthritis (OA), including extracellular matrix (ECM) degradation, inflammatory signaling, and metabolic dysregulation [12]. Given that OA fundamentally involves coordinated dysfunction of articular cartilage, subchondral bone, and synovial tissues, no single biomarker can adequately represent disease progression. Instead, biomarkers detected in synovial fluid, serum, and urine reflect overlapping aspects of tissue remodeling [12,13]. This biological complexity imposes a critical constraint on the development of next-generation biosensors. Effective monitoring requires platforms capable of multiplex detection and temporal resolution, rather than single-analyte, static measurements. For clarity, soluble biomarkers can be grouped into structural, inflammatory, and metabolic classes, each with distinct analytical and practical considerations.

### 3.1. Structural Biomarkers

Among soluble biomarkers, Structural biomarkers derived from cartilage ECM degradation are among the most widely studied [14,15]. Articular cartilage is primarily composed of type II collagen and proteoglycans, which provide mechanical strength and resilience. During OA progression, increased activity of degradative enzymes such as matrix metalloproteinases (MMPs) and aggrecanases leads to enzymatic cleavage of these matrix components releasing measurable fragments into synovial fluid and systemic circulation [16,17]. Key biomarkers such as the CTX-II and its urinary form (uCTX-II) are widely used indicators of collagen breakdown and have been correlated with disease severity [18]. Similarly, COMP, a non-collagenous ECM protein involved in collagen fibril stabilization, is also released during cartilage degradation and mechanical stress, serving as a marker of joint tissue damage [19]. Despite their clinical relevance, structural biomarkers present limitations for biosensing. They are not fully disease-specific and may originate from other connective tissues, leading to inter-patient variability. In addition, systemic sampling can dilute joint-specific signals. These constraints highlight the need for high selectivity, localized sampling, and multiplex detection strategies to improve diagnostic accuracy.

### 3.2. Inflammatory Biomarkers

Inflammatory mediators represent another critical class of soluble biomarkers that reflect active disease progression [20]. Increasing evidence shows that OA involves chronic low-grade inflammation rather than purely degenerative changes [16,21,22]. Pro-inflammatory cytokines such as interleukin-1β (IL-1β) and TNF-α disrupt cartilage homeostasis by activating catabolic signaling pathways [23], For example, IL-1β activates Nuclear Factor kappa-light-chain-enhancer of activated B cells (NF-κB) signaling, promoting the expression of matrix-degrading enzymes while suppressing repair mechanisms. Inflammatory signaling also induces secondary mediators such as nitric oxide and reactive oxygen species, further accelerating tissue damage [24]. Despite their strong biological relevance, these biomarkers present significant challenges from a sensing perspective. They are typically present at low concentrations and exhibit strong spatial and temporal variability within the joint. As a result, detection requires highly sensitive platforms with stable signal output and sufficient temporal resolution for continuous monitoring.

### 3.3. Metabolic Biomarkers

Complementary to structural and inflammatory markers, metabolic indicators provide additional insight into cellular activity within the osteochondral environment [25,26]. Articular cartilage operates under hypoxic conditions and relies primarily on anaerobic glycolysis [27]. Under pathological conditions, altered chondrocyte metabolism leads to changes in glucose consumption and increased lactate production within the joint environment [28,29]. These metabolic shifts reflect cellular stress and altered energy homeostasis and can therefore serve as indirect indicators of cartilage dysfunction. Although these biomarkers are less specific than structural indicators, they reflect cellular stress and energy imbalance. When interpreted alongside structural and inflammatory markers, they provide a more complete picture of disease progression [30]. This further supports the need for multiplex biosensing strategies capable of integrating multiple biological signals.

### 3.4. Implications for Biosensor Development

The characteristics of soluble biomarkers introduce several practical challenges for biosensor development. Biomarker concentrations vary depending on the sampling matrix: synovial fluid provides direct insight into intra-articular processes but is difficult to access, whereas blood and urine are more accessible but dilute joint-specific signals. In addition, biomarker levels fluctuate over time, reflecting dynamic biological activity rather than static disease states.

These factors impose key design requirements. Biosensors must achieve low detection limits, maintain selectivity in complex biological environments, and operate reliably under dynamic physiological conditions. Importantly, the temporal variability of biomarker expression necessitates continuous or longitudinal monitoring strategies.

Collectively, no single biomarker captures the full complexity of OA. Structural, inflammatory, and metabolic markers each reflect distinct aspects of disease progression, reinforcing the need for multiplex detection platforms. A structured comparison of biomarker–sensor relationships, analytical performance, and translational relevance is provided in Section 5 highlighting the diversity of sensing approaches and their current limitations.

### 3.5. EV-Mediated Pathophysiology and Cargo Profiling

Beyond freely soluble analytes, extracellular vesicles (EVs) have emerged as important molecular reservoirs for monitoring osteochondral degeneration and osteoarthritis (OA). These membrane-bound nanoparticles are released by chondrocytes, synoviocytes, and immune cells, mediating intercellular communication through the transfer of proteomic, lipidomic, and transcriptomic cargo [31,32]. Because their composition reflects the biochemical and inflammatory state of the joint, EVs provide localized molecular information with potentially greater biological specificity than freely circulating soluble biomarkers [32].

EVs represent a fundamental paradigm change in diagnostics. While soluble molecules often reflect fragmented biochemical processes, EVs encapsulate integrated molecular information within a single structure, providing a more specific representation of disease processes. This distinction has important implications for transition in biosensor design, shifting the focus from single-analyte detection toward the analysis of complex nanoscale entities.

Unlike soluble biomarkers, which often represent isolated biochemical processes, EVs encapsulate multidimensional molecular information within a single nanoscale structure. This shifts biosensor development from single-analyte detection toward integrated molecular profiling. EV populations are inherently heterogeneous, differing in size, cellular origin, and biogenesis pathways [33,34,35,36]. Exosomes (30–150 nm) originate from the endosomal pathway, whereas microvesicles are generated through membrane budding [37]. Their molecular cargo reflects key pathological processes associated with OA, including extracellular matrix degradation, inflammatory signaling, and cellular stress responses [38].

EVs derived from chondrocytes and synovial cells contain proteins, enzymes, and microRNAs associated with cartilage remodeling, inflammatory pathways, and chondrocyte apoptosis [37,39]. Because this cargo is selectively packaged rather than passively released, EV composition may provide a more biologically relevant representation of disease progression than freely circulating soluble biomarkers. In addition, the lipid bilayer protects encapsulated molecules from enzymatic degradation, improving molecular stability and detection reproducibility [39,40,41,42].

Despite these advantages, EV heterogeneity and the difficulty of isolating disease-specific vesicle populations remain major analytical challenges [33]. These limitations necessitate highly selective and high-resolution detection strategies capable of resolving complex nanoscale biological signals.

### 3.6. Implications and Challenges for the Development of EV Biosensors

Although EV-associated biomarkers offer significant biological advantages, their integration into biosensing platforms presents substantial analytical and engineering challenges [41]. Conventional isolation methods, including ultracentrifugation [43,44], size-exclusion chromatography, and immunoaffinity capture [45,46], are often labor-intensive and poorly suited for rapid diagnostics or continuous monitoring.

A major limitation arises from the nanoscale size and structural complexity of EVs [47]. Unlike soluble biomarkers, EV detection requires sensing platforms capable of nanometer-scale resolution while maintaining high selectivity in complex biological environments such as synovial fluid. In addition, nonspecific adsorption and surface fouling can impair vesicle capture and signal transduction, reducing sensitivity and reproducibility [41]. Current biosensor architectures are also largely optimized for soluble targets and often lack the ability to distinguish intact vesicles from free proteins or other nanoscale particles present in biological samples.

Addressing these limitations will require advances in biosensor design incorporating affinity-based capture interfaces, nanostructured materials, and microfluidic enrichment systems to improve vesicle isolation and detection efficiency. Integration of multiple sensing modalities may further enable simultaneous analysis of vesicle concentration, surface markers, and internal cargo. However, achieving selective EV capture together with long-term operational stability remains challenging due to nonspecific adsorption, intrinsic EV heterogeneity, and the demands of sustained operation within dynamic intra-articular environments.

From a translational perspective, EV-based biosensing may overcome several limitations associated with soluble biomarker detection by providing more stable and multidimensional molecular information. Nevertheless, realizing this potential will require improved nanoscale sensing technologies, more effective vesicle discrimination strategies, and validation in clinically relevant biological samples. Collectively, EV-associated biomarkers expand osteochondral biosensing from single-analyte detection toward integrated molecular profiling at the nanoscale. Future biosensor platforms may benefit from combining EV detection with multiplex soluble biomarker sensing strategies [48] to enable more comprehensive monitoring of osteochondral degeneration.

## 4. Joint Environment and Sampling Matrices

The biological sampling environment is a critical determinant of biosensor performance in osteochondral degeneration. A wide range of matrices has been used in biosensor studies, including synovial fluid [10,29,49,50,51], serum [52,53,54], blood, urine [52,55,56,57,58], artificial control solutions [59,60,61,62,63,64], in vitro cartilage models [65], organ-on-chip platforms [28], and in vivo experimental systems [66]. While this diversity reflects rapid technological development, it also highlights a key limitation: the lack of standardized and physiologically relevant validation frameworks. As a result, biosensor performance demonstrated in simplified environments often does not translate to complex clinical conditions.

Among available matrices, synovial fluid provides the most direct representation of intra-articular biochemical processes because it is in direct contact with cartilage, synovial membrane, and subchondral bone. It contains biomarkers associated with extracellular matrix degradation, inflammation, and metabolic stress. This makes it the most physiologically relevant matrix for osteoarthritis monitoring. However, its clinical use is limited by the need for invasive collection procedures such as arthrocentesis, restricting its suitability for continuous monitoring. This creates a fundamental trade-off between physiological relevance and sampling accessibility. To address these limitations, many studies have explored more accessible matrices such as blood, serum, and urine. These enable minimally invasive or non-invasive sampling and are therefore attractive for clinical diagnostics. For example, urinary C-terminal telopeptide of type II collagen (uCTX-II) [52,55,57,61,62] has been widely investigated as a biomarker of cartilage degradation, with multiple biosensing platforms including interdigitated electrode immunosensors [57,61], optical colorimetric systems [52,62], and electrochemical self-assembled monolayer sensors [55] demonstrating high analytical sensitivity. Similarly, COMP [50,53,56] has been detected in serum and synovial fluid using electrochemical, capacitive, and QCM biosensors. However, these systemic matrices introduce significant analytical challenges. Biomarkers originating from the joint are often diluted through circulation and metabolic clearance, reducing detection sensitivity. In addition, signals from other tissues can confound interpretation, limiting specificity.

Beyond structural biomarkers, a range of enzymatic, inflammatory, and metabolic biomarkers have been investigated across different sampling environments. MMP-3 [10,59] and collagenase [66] have been detected as indicators of extracellular matrix degradation, while inflammatory cytokines such as interleukin-1β (IL-1β) [49] and TNF-α [10] have been measured using fiber-optic plasmon resonance and other optical sensing platforms. Although these biomarkers are highly relevant, their low concentrations and spatial heterogeneity present challenges for reliable detection. Metabolic markers such as glucose [28] and lactate [28,29] have been studied in cartilage-on-chip and microphysiological systems, enabling controlled investigation of chondrocyte activity under physiologically relevant conditions. Similarly, electrochemical and transistor-based sensors have demonstrated the feasibility of detecting nitric oxide in both in vitro and in vivo models [65,67] demonstrating the feasibility of monitoring oxidative stress within the joint environment.

Emerging biomarkers such as cartilage acidic protein 1 (CRTAC1) [54] and hyaluronic acid [60] further expand the scope of detectable molecular targets. CRTAC1 has been measured using single-walled carbon nanotube field-effect transistor biosensors, whereas hyaluronic acid has been detected using phosphorescent optical platforms. While these advances highlight the growing diversity of biosensing applications, they also reinforce the need for platforms capable of operating in complex and heterogeneous biological environments. Importantly, these considerations are not merely contextual but directly influence the feasibility of implantable and intra-articular biosensing systems. Sensor performance is fundamentally constrained by matrix complexity, accessibility, and physiological variability. These factors must therefore be incorporated into biosensor design and validation strategies, as further discussed in subsequent sections.

## 5. Biosensing Technologies for Osteochondral Monitoring

The transition from static biochemical assays to real-time osteochondral monitoring requires biosensing platforms capable of operating in complex joint environments. Current approaches can be broadly categorized into electrochemical, optical, and piezoelectric systems. While each modality offers distinct advantages, their clinical utility ultimately depends on performance under physiologically relevant conditions, including stability, miniaturization, and intra-articular compatibility.

Accordingly, these platforms are evaluated not only in terms of analytical sensitivity but also with respect to implantability, operational stability, and suitability for intra-articular applications. A comparative summary of representative biosensing platforms, including key performance metrics and translational considerations, is provided in the following subsections.

### 5.1. Electrochemical Biosensors

Electrochemical biosensors represent a promising class of diagnostic platforms for osteochondral monitoring, offering a rare combination of high sensitivity, potential for miniaturization, and inherent compatibility with complex biological matrices [28,50,53,54,55,63,65,67]. By converting biochemical recognition events into discrete electrical signals via amperometric [28,65,67], potentiometric [53,63], or impedimetric transduction [55], these platforms bypass the optical interference common in opaque media like synovial fluid. This capability positions electrochemical systems as the promising option for point-of-care (POC) diagnostics and intra-articular sensing, where traditional fluorometric or colorimetric assays often fail.

The versatility of electrochemical architectures is evidenced by their success in detecting a broad spectrum of OA-related analytes. For instance, impedimetric immunosensors utilizing interdigitated electrodes have achieved precise quantification of urinary CTX-II [55], while similar platforms have been optimized for the detection of structural proteins such as COMP [50,53] and C-terminal telopeptide of type I collagen (CTX-I) [63]. Beyond structural markers, electrochemical microsensors have enabled the real-time monitoring of nitric oxide (NO) within cartilage models [65], providing high-resolution temporal data on inflammatory kinetics that are unattainable through endpoint molecular assays.

**Figure 1:**
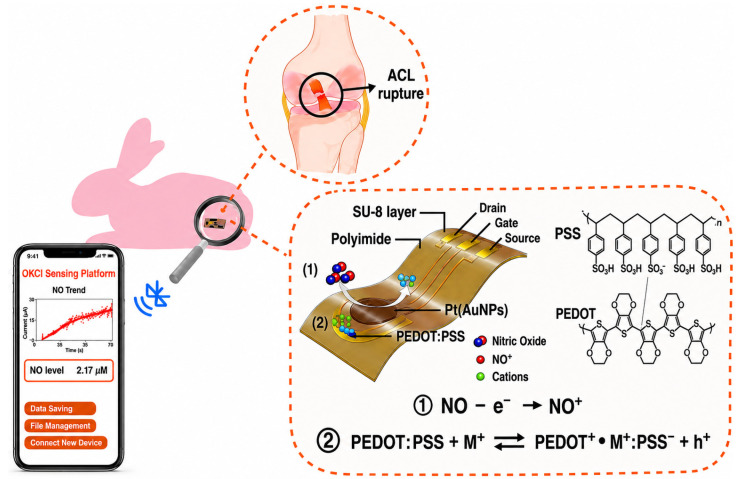
Conceptual representation of an implantable electrochemical biosensor for intra-articular nitric oxide (NO) monitoring. The schematic depicts the operational principle of a flexible organic electrochemical transistor (OECT)-based platform, highlighting direct electrochemical transduction and wireless signal integration for real-time sensing in the joint environment. This design underscores the potential of electrochemical systems for implantable, continuous monitoring due to their compatibility with miniaturized electronics and function in complex biological media. Adapted and modified from Deng et al., 2022 (CC BY 4.0) [67].

Recent breakthroughs in organic electrochemical transistors (OECTs) and field-effect transistors (FETs) further underscore the translational momentum of this field. Deng et al. demonstrated a flexible, OECT-based biosensor capable of continuous, nanomolar-scale NO monitoring (~3 nM) directly within the synovial fluid of animal models [67]. Such systems, characterized by stable signal output and mechanical flexibility, represent an important step toward wearable or implantable joint diagnostics (Figure 1). Single-walled carbon nanotube (SWCNT) FETs have expanded the detection repertoire to emerging biomarkers like CRTAC1 [54], while the integration of electrochemical sensors into cartilage-on-chip systems has facilitated the longitudinal tracking of metabolic fluxes, such as glucose and lactate [28].

Despite these advantages, electrochemical biosensors remain limited by electrode biofouling and signal drift, which can compromise long-term stability in biological environments. Addressing these challenges is essential for reliable in vivo and implantable applications.

### 5.2. Optical and Fiber-Optic Biosensors

Optical biosensors represent one of the most widely investigated platforms for osteoarthritis biomarker detection [10,29,49,52,57,59,60,61,62,68]. These systems rely on optical transduction mechanisms such as fluorescence emission [52,61], absorbance variation [57,62], refractive index modulation [10,49,59], and phosphorescence intensity changes [60] to detect biomolecular interactions.

Fiber-optic plasmon resonance platforms [10,49,59] have been extensively applied for detecting inflammatory cytokines including IL-1β [50] and TNF-α [10], as well as matrix-degrading enzymes such as MMP-3 [10,59]. For example, Chiang-Yue C. et al. demonstrated a fiber-optic particle plasmon resonance biosensor with detection limits of approximately 21 pg mL^−1^ for IL-1β [49], while Huang Y. et al. reported multiplex detection of TNF-α and MMP-3 with picogram-level sensitivity [10]. Optical biosensors have also been applied to detect structural biomarkers such as uCTX-II [52,57,61,62] and synovial components such as hyaluronic acid [60] and metabolic biomarkers such as lactate using flexible fiber-optic systems [29].

Recent advances in engineered nanomaterials, including chromium-doped zinc gallate nanoparticles and magnetic iron oxide nanostructures, have demonstrated improved optical stability, prolonged luminescence, enhanced magnetic responsiveness, and multifunctional imaging capabilities [69,70,71,72]. These developments further support the growing relevance of nanostructured materials in advanced biosensing and diagnostic platforms. Emerging nanopore biosensing systems additionally expand the scope of miniaturized sensing technologies by enabling rapid and high-throughput biomarker detection with minimal sample preparation requirements [73].

**Figure 2:**
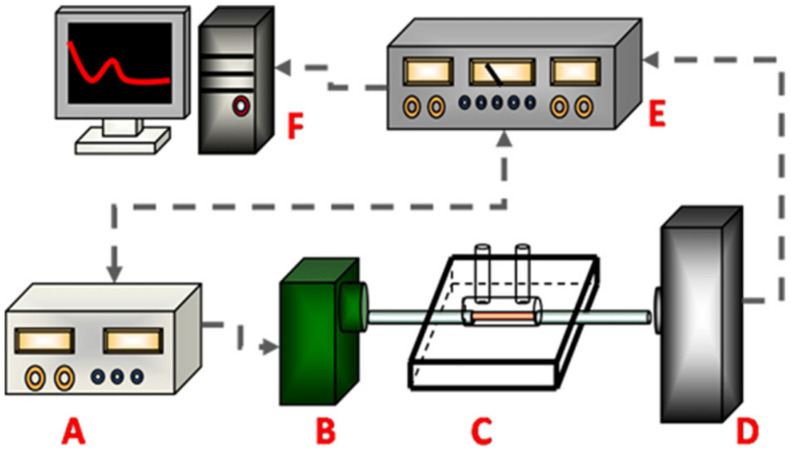
Conceptual representation of an optical Fiber Optic-Particle Plasmon Resonance (FO-PPR) biosensing system, highlighting the instrumentation requirements for signal generation and detection. The schematic illustrates the reliance on external light sources, photodetection units, and signal processing modules, emphasizing the complexity and bulkiness of optical platforms. This configuration underscores key translational limitations for intra-articular applications, including challenges in miniaturization, integration, and real-time deployment in the joint environment. Key components are indicated as follows: A—function generator; B—light-emitting diode; C—FO-PPR sensor chip; D—photodiode; E—lock-in amplifier; F—computer. Adapted from [59] with permission.

Despite their high analytical sensitivity, optical biosensors face important limitations for practical intra-articular implementation. Many systems require bulky instrumentation, external light sources, photodetectors, and precise optical alignment, restricting their portability and applicability outside laboratory environments (Figure 2) [59]. In addition, optical signal transmission may be affected by scattering and absorption in turbid biological fluids such as synovial fluid, reducing signal reliability [49]. These limitations continue to constrain the development of implantable or continuous optical biosensing systems despite their strong analytical performance.

### 5.3. Acoustic and Mass-Sensitive Biosensing Platforms

**Figure 3:**
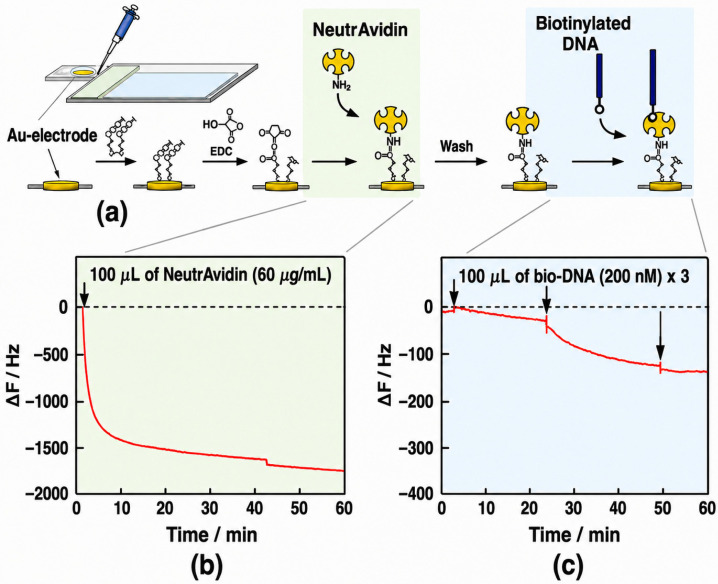
Conceptual representation of a piezoelectric (QCM)-based biosensing mechanism illustrating surface functionalization and analyte binding dynamics. The schematic highlights the sequential immobilization strategy and corresponding frequency shifts (Δ*F*) used for signal generation, demonstrating the high sensitivity of mass-based detection. However, this approach also reveals key limitations for intra-articular applications, including dependence on stable oscillatory conditions and susceptibility to mechanical perturbations in dynamic physiological environments. (**a**) Schematic illustration of DNA immobilization process on the QCM sensor surface. Time courses of a frequency change (Δ*F*), responding to bindings of (**b**) NeutrAvidin to the activated carbonic acids and (**c**) biotinylated DNA (10-mer) to the NeutrAvidin on the QCM sensor surface in each buffer solution: 10 mM HEPES-NaOH (pH 7.9), 0.2 M NaCl for the NeutrAvidin binding and 10 mM Tris-HCl (pH 8.0), 1 mM EDTA, 0.15 M NaCl for biotinylated DNA binding at 21 °C. The arrow in (**b**) indicates the injection time of the NeutrAvidin solution and arrows in (**c**) indicate the timing of each injection of the biotinylated DNA solution. i.e., 100 µL of bio-DNA (200 nM). Adapted and modified from Yoshimine et al., 2023 (Sensors), licensed under CC BY 4.0 [74].

Piezoelectric biosensors, particularly those based on quartz crystal microbalance (QCM) technology, have been explored for detecting osteoarthritis biomarkers through mass-sensitive detection [56,66,74] (Figure 3). These systems measure changes in resonance frequency resulting from biomolecular binding at the sensor surface, enabling highly sensitive detection of analytes such as collagenase [66] and COMP [56]. For instance, QCM immunosensors have demonstrated rapid and sensitive detection of COMP in urine, with frequency shifts of approximately 6 kHz at 50 ng mL^−1^ [56], while hydrogel-coated QCM platforms have been used to detect collagenase activity with nanomolar sensitivity (2 nM) [66]. These results highlight the strong analytical capabilities of QCM-based sensing.

However, the intrinsic operating principles of QCM pose substantial challenges for translation into osteochondral diagnostics. The requirement for stable mechanical oscillation of rigid quartz crystals renders these sensors acutely susceptible to environmental vibrations and fluctuating fluid dynamics. In the mechanically dynamic environment of a weight-bearing joint, maintaining the necessary frequency stability is technically prohibitive, often leading to poor signal-to-noise ratios. Furthermore, the reliance on bulky oscillation circuitry and precisely controlled experimental chambers inherently limits the miniaturization and implantability of QCM systems. Consequently, while QCM remains a powerful tool for ex vivo mechanistic studies and benchtop fluid analysis, its transition to wearable or intra-articular monitoring remains constrained by these fundamental mechanical sensitivities.

These figures illustrate representative sensing mechanisms across different biosensor platforms; however, the primary comparative and translational synthesis of these technologies is systematically presented in Section 5.4 and critically discussed in Section 7.

### 5.4. Overall Evaluation and Translational Perspective

While biosensor platforms demonstrate diverse analytical capabilities, their performance and clinical relevance vary depending on sensing modality, target biomarker, and detection environment. A comparative summary of key biosensing platforms is provided in Table 1, with particular emphasis on their suitability for intra-articular deployment, operational stability under physiological conditions, and overall translational readiness. While key metrics such as detection limits are widely reported, parameters including response time, linear range, and long-term stability are inconsistently documented across studies, limiting direct quantitative comparison. A detailed discussion of translational challenges and future directions is presented in Section 7.

## 6. Implantability and Bio-Integration

Transitioning from ex vivo assays to continuous intra-articular monitoring necessitates a fundamental evolution in sensor architecture, shifting focus from analytical performance to long-term biostability and mechanical compliance. The joint space represents a uniquely hostile environment for electronic integration, characterized by high-cycle mechanical loading, fluctuating pressures, and a robust foreign body response that can lead to fibrous encapsulation and sensor passivation. For a biosensor to remain clinically viable in situ, it must transcend simple miniaturization to achieve true biochemical and mechanical synergy with the host tissue through biocompatible encapsulation, advanced power management (including passive radio frequency and energy harvesting), and the development of flexible, low-modulus materials designed to mitigate tissue-sensor mismatch. Recent advances in flexible electrochemical biosensors based on two-dimensional nanomaterials have further highlighted the growing potential of mechanically compliant and miniaturized sensing systems for wearable and implantable biosensing applications [75].

A critical evaluation of current platforms reveals that electrochemical systems offer the most viable pathway for implantation. Their signal transduction is inherently compatible with opaque biological fluids and can be integrated into miniaturized, flexible electronics. While architectures such as amperometric microsensors [28,65,67], interdigitated electrodes [53,63], and field-effect transistors [54] are compatible with wireless data transmission, only Deng et al. have demonstrated successful intra-articular functionality using a flexible organic electrochemical transistor for continuous nitric oxide monitoring in rabbit models [67]. Despite this progress, most electrochemical sensors remain at the proof-of-concept stage, facing persistent challenges such as electrode biofouling, signal drift, and mechanical mismatch. As noted by Belcastro et al., robust interfacial engineering and polymer-based antifouling strategies are essential to suppress interference and ensure signal reproducibility in vivo [65].

In contrast, optical and piezoelectric platforms face significant translational bottlenecks. Optical biosensors [10,29,49,52,57,59,60,61,62,68], though dominant in the literature, typically require external light sources, photodetectors, and precise optical alignment [59]. Signal attenuation due to scattering in turbid synovial fluid further limits their reliability for continuous in vivo monitoring. Similarly, piezoelectric (QCM) sensors [56,66] exhibit the lowest implantability potential; the requirement for stable mechanical oscillation of rigid quartz crystals is fundamentally incompatible with the dynamic, weight-bearing motion of the knee.

Collectively, these observations indicate that implantability remains a major bottleneck across all biosensor platforms. Electrochemical systems currently offer the most promising pathway toward intra-articular monitoring, yet even these technologies face substantial challenges related to long-term stability, mechanical compliance, and biofouling resistance. Achieving reliable implantable biosensing will require advances in flexible and biocompatible materials, robust antifouling surface chemistries, and stable signal transduction mechanisms under cyclic mechanical loading.

Beyond hardware considerations, implantable biosensors will also generate continuous streams of biochemical data from the joint environment. The integration of such systems with digital data-processing frameworks, including machine-learning-assisted signal interpretation, may enable real-time analysis of intra-articular biomarker dynamics and improve clinical decision-making.

Ultimately, implantability represents the critical transition point between analytical biosensor performance and clinical applicability. Addressing the mechanical, biological, and analytical constraints of intra-articular environments will be essential for transforming current biosensor technologies into reliable tools for continuous monitoring of osteochondral degeneration and osteoarthritis. Considerations related to scalability, manufacturability, and clinical translation are further discussed in Section 7.

## 7. Clinical Landscapes

Given the stability and bio-integration constraints identified in Section 5, a critical observation emerges: despite significant analytical advances, no biosensor designed for the direct biochemical detection of osteoarthritis-related biomarkers has yet progressed to formal human clinical trials. The platforms reviewed herein, including electrochemical [28,50,53,54,55,63,65,67], optical [10,29,49,52,57,59,60,61,62,68], and piezoelectric [56,66] systems, have been evaluated primarily in vitro or ex vivo, with only a single study demonstrating in vivo functionality in an animal model [67].

This lack of clinical translation does not reflect insufficient analytical performance. Many platforms demonstrate exceptional sensitivity for key biomarkers, including CTX-II [55,57,61,62], COMP [50,53,56], MMPs [10,59], nitric oxide [65,67,68], glucose, and lactate [28,29]. Rather, the primary limitations arise from unresolved challenges related to long-term stability, biofouling resistance, and mechanical compatibility within the dynamic intra-articular environment. These constraints, discussed in Section 4 and Section 5, remain the major barriers preventing progression from laboratory validation to clinical application.

In addition to technical limitations, regulatory and biocompatibility related requirements for implantable devices impose stringent safety and performance standards that current platforms have yet to satisfy under physiologically relevant conditions. Translation into clinical use requires comprehensive preclinical validation, biocompatibility assessment, and long-term safety evaluation under regulatory frameworks such as those of the U.S. Food and Drug Administration (FDA) and the European Medicines Agency (EMA). Emerging microfluidic and organ-on-chip validation systems may help reduce reliance on animal testing while improving physiologically relevant preclinical assessment, potentially accelerating regulatory evaluation pathways. Additionally, implantable biosensors must demonstrate not only analytical accuracy but also mechanical reliability, electrical safety, and resistance to long-term degradation in vivo.

Established implantable technologies, such as continuous glucose monitoring (CGM) systems, provide a relevant benchmark for clinical translation, having achieved regulatory approval based on minimally invasive design, robust encapsulation strategies, and validated long-term performance in clinical settings. In contrast, current osteochondral monitoring approaches, including clinical trials such as NCT05222503 and NCT06416332, remain largely limited to wearable motion sensors and digital telemonitoring, which provide biomechanical data but do not capture the underlying biochemical dynamics of disease progression.

Collectively, these observations highlight a fundamental gap between analytical capability and clinical deployment. Bridging this gap will require biosensing platforms that maintain signal fidelity within the joint environment while meeting regulatory standards for safety, stability, and long-term operation. This unmet need motivates the design strategies outlined in the following section.

## 8. Discussion and Future Perspectives

Osteoarthritis remains a major global health burden, while current diagnostic approaches based on imaging and clinical assessment remain limited in detecting early molecular changes within the joint [76,77]. In this context, biosensors offer promising opportunities for real-time detection of biomarkers associated with cartilage degradation, inflammation, and metabolic dysregulation. Across the electrochemical [28,50,53,54,55,63,65,67], optical [10,29,49,52,57,59,60,61,62,68], and piezoelectric systems [56,66] reviewed, substantial advances have been achieved in analytical sensitivity and biomarker specificity. Optical systems demonstrate excellent sensitivity and multiplexing capability, as reported by Huang et al. [10,49,59], whereas electrochemical platforms offer advantages for intra-articular applications due to their compatibility with the joint microenvironment and suitability for miniaturization [67].

Tengbo Lv et al. reported ultra-sensitive electrochemical detection of CRTAC1 in serum with a detection limit of 0.2 fg/mL, highlighting the strong potential of electrochemical platforms for intra-articular sensing [54]. Because these systems rely on direct electrical transduction rather than optical signal propagation, they are less susceptible to interference from the turbid and highly scattering nature of synovial fluid and do not require external optical alignment, enabling more stable operation under physiological conditions. Despite these advances, most biosensing platforms remain confined to in vitro or ex vivo validation, with only two studies by Deng et al. and Li et al. demonstrating in vivo functionality [29,67] and none progressing to human clinical trials. A major limitation is the lack of standardized testing conditions, with studies variably performed in buffer systems [61,62,63,66], synovial fluid [10,29,49,50,59], or limited clinical samples [52,55], making direct comparison difficult. In addition, parameters such as response time and long-term stability are inconsistently reported across studies. These limitations highlight the need for physiologically relevant validation platforms, including microfluidic joint models and controlled synovial fluid mimics.

Long-term operational stability remains one of the principal barriers to implantable intra-articular biosensing. Mechanical mismatch, cyclic loading, biofouling, fibrosis, enzymatic degradation, corrosion, and calibration drift can collectively reduce signal reliability and shorten device lifespan. In particular, the protein-rich composition of synovial fluid promotes rapid nonspecific adsorption onto sensor surfaces, impairing signal transduction and accelerating instability under mechanical strain [78]. Surface modification strategies, including zwitterionic coatings, polyethylene glycol (PEG) layers, and hydrophilic polymer interfaces, have shown promise for mitigating these effects and improving long-term stability [79,80]. Further advances in mechanically compliant materials, flexible architectures, and encapsulation strategies will be essential for durable intra-articular operation.

Another major limitation is the predominance of single-analyte detection despite the multifactorial nature of osteoarthritis. Most currently reported platforms focus on isolated biomarkers, with only one study demonstrating true multiplexed detection [10]. Signal cross-reactivity, limited surface functionalization capacity, and interference from complex synovial matrices remain major barriers to multiplexed sensing. Future multiplexed systems will likely require microarray-based electrode architectures, spatially separated sensing domains, and integrated microfluidic handling to enable simultaneous detection of multiple biomarkers within complex synovial environments.

Scalability and regulatory considerations further constrain clinical implementation. Many biosensor platforms rely on complex nanofabrication and surface functionalization processes that limit reproducibility and increase manufacturing cost. Implantable systems must additionally satisfy stringent requirements for long-term biocompatibility, mechanical reliability, and safety. Regulatory approval pathways for implantable devices therefore require extensive validation under physiologically relevant conditions, substantially prolonging development timelines.

Future progress will require integrated system-level approaches that combine advanced sensing architectures with physiologically relevant validation and data-driven analysis. Recent developments in amplification-assisted nanopore biosensing, click-chemistry-enabled sensing, single-molecule detection, and enrichment-based signal discrimination strategies have demonstrated ultrasensitive biomarker detection in complex biological fluids [81,82,83]. Similarly, triggerable biomaterial-based nanoplatforms and extracellular vesicle (EV)-based sensing systems continue to expand opportunities for multifunctional and high-specificity biosensing applications [84,85]. Physiologically relevant validation systems, including cartilage-on-chip and microfluidic joint models, will also play an important role in bridging the gap between benchtop testing and in vivo application.

The transition toward continuous monitoring will further require advanced computational frameworks capable of integrating longitudinal biomarker data. Machine learning approaches may improve signal interpretation through noise reduction, drift correction, and integration of multimodal biosensor outputs [86,87]. In future clinical workflows, implantable and wearable biosensors could complement existing diagnostic modalities such as MRI, biochemical assays, and digital health platforms by enabling continuous monitoring of joint-specific biochemical changes and supporting more personalized assessment of osteoarthritis progression.

**Figure 4:**
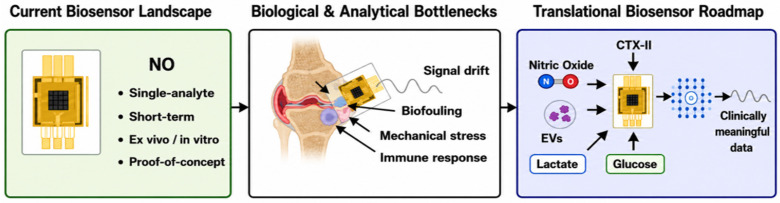
Original conceptual framework developed in this review, illustrating the translational pathway from current biosensor capabilities to clinically viable intra-articular sensing systems. The left panel summarizes the present landscape, dominated by single-analyte (such as Nitric Oxide-NO), short-term, proof-of-concept studies conducted primarily under in vitro or ex vivo conditions. The central panel highlights key biological and analytical barriers, including biofouling, mechanical stress, immune response, and signal drift. The right panel outlines future design priorities, emphasizing multiplex biomarker detection (structural, inflammatory, metabolic, and extracellular vesicle-associated), mechanically compliant and implantable sensor architectures, and integration with data-driven analytical frameworks to enable clinically meaningful monitoring of osteoarthritis progression. Created with BioRender.com.

From a translational perspective, the development of biosensing systems can be understood within a technology readiness level (TRL) framework [88]. To the best of our knowledge, most currently reported biosensors remain at the proof-of-concept stage (TRL 1–3), while only a limited number have progressed to validation under physiologically relevant conditions (TRL 4–6). Advanced stages (TRL 7–9) require integration into implantable formats, long-term in vivo validation, and compliance with regulatory standards before clinical deployment can be achieved. The current state-of-the-art is largely limited to validation under physiologically relevant conditions (TRL 4–6), although continued advances in implantable biosensing are expected to accelerate progression toward higher TRLs and eventual clinical deployment. A roadmap for achieving this is provided in Figure 4.

This review highlights a clear shift in the field from analytical feasibility toward robust and clinically deployable biosensing systems. Achieving reliable intra-articular monitoring will ultimately depend on the successful integration of multiplexed sensing, mechanically adaptive materials, antifouling stability, physiologically relevant validation, and data-driven analytical frameworks.

## 9. Conclusions

Osteoarthritis remains a major clinical challenge, particularly due to limitations in early detection. Biosensing technologies offer a promising approach for the sensitive and real-time detection of disease-related biomarkers. This review demonstrates that, despite significant advances in biosensor sensitivity, the primary barrier to clinical translation lies in achieving stable and reliable operation within the mechanically dynamic and biochemically complex intra-articular environment. Current platforms remain largely confined to proof-of-concept validation, highlighting a persistent gap between analytical performance and real-world applicability.

Bridging this gap requires a shift toward integrated system design that accounts for biological, mechanical, and operational constraints. Key priorities include multiplexed biomarker detection, the development of mechanically compliant and biocompatible sensor architectures, the implementation of robust antifouling strategies for long-term stability, validation under physiologically relevant and in vivo conditions, and the integration of data-driven approaches for real-time signal interpretation. Advancing along these directions will enable the transition from experimental biosensing platforms to clinically deployable systems for continuous intra-articular monitoring and improved management of osteoarthritis.

## Figures and Tables

**Table 1:** A structured comparison linking key biomarkers with corresponding sensing modalities, detection mechanisms, analytical performance (including detection limits), response time, linear range, stability, sample matrices, and translational relevance. Where available, these performance parameters are reported to enable comparative evaluation across platforms.

S/N	Biomarker	Biomarker Class	Sensor Type	Detection Mechanism	Detection Matrix	Limit of Detection	Response Time	Linear Range	Stability	Evidence Level	Key Limitation	Translational Potential	Ref.
1	Nitric oxide (NO)	Metabolic	Electrochemical microsensor (Pt/PPD)	Amperometric	Chondrocyte culture (DMEM)	0.2 µM	Not reported	2–100 µM	48 h only	In vitro	Interference in complex media	Moderate–High	[65]
2	COMP	Structural	FMGC optical biosensor	Sandwich immunoassay	Serum, synovial fluid	0.8 ng/mL	~20 min	4–128 ng/mL	Not reported	Ex vivo	Requires microscopy	High	[50]
3	uCTX-II	Structural	IDE electrochemical immunosensor	Impedance (immuno-binding)	Urine (buffer-tested)	10–100 pM	Not reported	10 pM–100 nM	Not reported	In vitro	Limited real-sample validation	Moderate–High	[55]
4	COMP fragments	Structural	ELISA	Sandwich immunoassay	Serum	Not reported	Not reported	10–100 ng/mL	Coefficient of variation only	In vitro–In vivo	Lab-based, no POCT	High	[53]
5	CRTAC1	Structural	SWCNT-FET biosensor	Label-free electrical	Serum	0.2 fg/mL	~4 h	up to 3 µg/mL	repeatability	In vitro + ex vivo	Fabrication complexity	Very High	[54]
6	CTX-I	Structural	Electrochemical immunosensor	Impedance	Buffer	50 ng/mL	~0.6 s	up to ~5 mM	good (drift studied)	In vitro	No clinical validation	Low–Moderate	[63]
7	Glucose, Lactate	Metabolic	Enzymatic electrochemical	Amperometric (GOx/LOx)	Cartilage-on-chip	~1 mM	Not reported	3 nM–100 µM	strong (10 days)	In vitro	Not disease-specific	Moderate–High	[28]
8	Nitric oxide (NO)	Metabolic	OECT biosensor	Electrochemical + transistor amplification	In vivo joint (rabbit)	~3 nM	Not reported	3 nM–100 µM	strong (10 days + in vivo)	In vivo	Requires implantation	High	[67]
9	TNF-α, MMP-3	Inflammatory	Optical LSPR biosensor	Plasmonic immunoassay	Synovia l fluid	pg/mL range	~290 s	very wide	short-term only	In vitro + ex vivo	Optical setup complexity	High	[10]
10	IL-1β	Inflammatory	FOPPR optical biosensor	LSPR immunoassay	Synovial fluid	21 pg/mL	~270 s	0.05–10 ng/mL	Not reported	In vitro + ex vivo	Single-analyte detection	High	[49]
11	ADMA, Lactate	Metabolic	Optical fiber biosensor	Optical signal modulation	Synovial fluid (in vivo)	Not reported	real-time (no value)	Not reported	in vivo only	In vivo	No human validation	High	[29]
12	CTX-II (uCTX-II/sCTX-II)	Structural	FMGC optical biosensor	Competitive/sandwich fluorescence	Urine, serum	~0.1 ng/mL	~70 min	clinical range	Coefficient of variation only	In vitro + ex vivo	Semi-quantitative	Moderate–High	[52]
13	uCTX-II	Structural	Smartphone optical biosensor	Colorimetric immunoassay	Artificial urine	Not reported	~70 min	0–10 ng/mL	short-term	In vitro	No real clinical validation	High	[57]
14	Hyaluronic acid	ECM	Phosphorescent optical sensor	RTP-based detection	Serum	0.03 µg/mL	~15 min	0.08–2.8 mg/mL	Relative standard deviation only	In vitro + ex vivo	Non-specific biomarker	Moderate–High	[60]
15	uCTX-II	Structural	Smartphone illumination sensor	Optical attenuation	Buffer	~0.2 ng/mL	~1.5–2 h	0–5 ng/mL	good (COV)	In vitro	Light dependency	High	[61]
16	uCTX-II	Structural	DIY optical biosensor	Colorimetric immunoassay	Buffer	~0.3 ng/mL	~10 min/~2 h	1.3–10 ng/mL	good	In vitro	Manual setup	Moderate–High	[62]
17	MMP-3 (model systems)	Inflammatory	FO-PPR biosensor	LSPR modulation	Synovial fluid	pM range	~5 min	log-linear	good	In vitro + ex vivo	Variable sensitivity	High	[59]
18	COMP	Structural	QCM biosensor	Mass-sensitive (frequency)	Urine	Not reported	~25 min	1–200 ng/mL	good	Ex vivo	Lower sensitivity	Moderate–High	[56]
19	Collagenase (MMP-1)	Inflammatory	QCM hydrogel biosensor	Enzymatic degradation	Buffer	2 nM	<10 min	2–2000 nM	weak	In vitro	No clinical validation	Moderate	[66]
20	Nitric oxide (NO)	Metabolic	SWCNT optical biosensor	Fluorescence quenching	Cells, tissue	~0.1 µM	seconds (real-time)	12.5–200 µM	excellent (30 days)	In vitro + ex vivo	Optical complexity	High	[68]

Note: Response time, linear range, and stability data are reported where available. Variability in reporting standards across studies limits direct quantitative comparison of these parameters. Abbreviations used in the table are as follows: Pt/PPD—Platinum electrode coated with a thin film of Poly(o-phenylenediamine), DMEM—Dulbecco’s Modified Eagle Medium, FMGC—Fluoro-Microbeads Guiding Chip, ELISA—Enzyme-Linked Immunosorbent Assay, POCT—Point-of-Care Testing, Gox—Glucose Oxidase, Lox—Lactate Oxidase, ADMA—Asymmetric Dimethylarginine, sCTX-II—serum C-terminal cross-linked telopeptide of type II collagen, MMP1—Matrix Metalloproteinase-1, LSPR—Localized Surface Plasmon Resonance, RTP—Real-time Transport Protocol.

## Data Availability

No new data was created.
